# Recent Developments Toward Integrated Metabolomics Technologies (UHPLC-MS-SPE-NMR and MicroED) for Higher-Throughput Confident Metabolite Identifications

**DOI:** 10.3389/fmolb.2021.720955

**Published:** 2021-09-02

**Authors:** Rajarshi Ghosh, Guanhong Bu, Brent L. Nannenga, Lloyd W. Sumner

**Affiliations:** ^1^ Division of Biochemistry, University of Missouri, Columbia, MO, United States; ^2^ MU Metabolomics Center, University of Missouri, Columbia, MO, United States; ^3^ Christopher S. Bond Life Sciences Center, University of Missouri, Columbia, SC, United States; ^4^ Interdisciplinary Plant Group, University of Missouri, Columbia, SC, United States; ^5^ Chemical Engineering, School for Engineering of Matter, Transport & Energy, Arizona State University, Tempe, AZ, United States; ^6^ Center for Applied Structural Discovery, The Biodesign Institute, Arizona State University, Tempe, AZ, United States

**Keywords:** integrated metabolomics, UHPLC-MS-SPE, NMR, MicroED, metabolite identification

## Abstract

Metabolomics has emerged as a powerful discipline to study complex biological systems from a small molecule perspective. The success of metabolomics hinges upon reliable annotations of spectral features obtained from MS and/or NMR. In spite of tremendous progress with regards to analytical instrumentation and computational tools, < 20% of spectral features are confidently identified in most untargeted metabolomics experiments. This article explores the integration of multiple analytical instruments such as UHPLC-MS/MS-SPE-NMR and the cryo-EM method MicroED to achieve large-scale and confident metabolite identifications in a higher-throughput manner. UHPLC-MS/MS-SPE allows for the simultaneous automated purification of metabolites followed by offline structure elucidation and structure validation by NMR and MicroED. Large-scale study of complex metabolomes such as that of the model plant legume Medicago truncatula can be achieved using an integrated UHPLC-MS/MS-SPE-NMR metabolomics platform. Additionally, recent developments in MicroED to study structures of small organic molecules have enabled faster, easier and precise structure determinations of metabolites. A MicroED small molecule structure elucidation workflow (e.g., crystal screening, sample preparation, data collection and data processing/structure determination) has been described. Ongoing MicroED methods development and its future scope related to structure elucidation of specialized metabolites and metabolomics are highlighted. The incorporation of MicroED with a UHPLC-MS/MS-SPE-NMR instrumental ensemble offers the potential to accelerate and achieve higher rates of metabolite identification.

## Introduction

Metabolomics is the large-scale profiling of metabolites and it has now been applied to a multitude of biological systems. Metabolomics has seen exponential growth over the past 2 decades mostly due to continual technology advancements in analytical instrumentation and computational tools. The discipline continues to play a key role in understanding metabolism, the elucidation of novel gene functions and the discovery of biomarkers (Sumner et al., 2015; Beger et al., 2020; Nakabayashi and Saito, 2020). In spite of the overwhelming success of metabolomics, the large-scale confident identification of metabolites continues to be a major challenge. In most published metabolomics studies, typically 20% or less of the spectral features obtained from mass spectrometry (MS) or nuclear magnetic resonance (NMR) are confidently identified (Blaženović et al., 2018). The primary approach to metabolite identification includes spectral matching with data from authentic standards hosted in mass spectral databases (e.g., HMDB, MassBank, NIST, METLIN etc. (Stein and Scott, 1994; Smith et al., 2005; Horai et al., 2010; Wishart et al., 2018)) and NMR databases (e.g., BMRB, COLMAR etc. (Ulrich et al., 2008; Bingol et al., 2015)). This approach offers a confident and fast way to annotate compounds (Blaženović et al., 2018). Currently, chemical structure databases such as PubChem contain hundreds of millions of compounds but only 1-2 million of these compounds are of biological relevance and only a fraction of these compounds are available as authentic standards or included in the spectral databases. Hence an overwhelming number of metabolites are not represented in metabolomics spectral databases resulting in poor rates of metabolite identification. Additional *in silico* computational tools (e.g., MetFragCL, CFM-ID, MAGMa+, MS-FINDER etc. (Allen et al., 2014; Ruttkies et al., 2016; Tsugawa et al., 2016; Verdegem et al., 2016; Djoumbou-Feunang et al., 2019)) can exploit the content of structural databases for alternative metabolite identification capabilities (Blaženović et al., 2017; Wolfender et al., 2019) and *in silico* predicted spectral databases have been developed from the approximately 100 million known compounds in PubChem and ChemSpider (Milman and Zhurkovich, 2017). The use of molecular networking (e.g., GNPS (Wang et al., 2016)) to cluster compounds based on mass spectral fragmentation similarity followed by putative annotation has also gained popularity in recent years. Unfortunately, it is difficult to unambiguously identify compounds based solely on molecular networking data. Overall, the growth and expansion of authentic spectral databases continue to be slow and there is an urgent need to explore and advance other strategies to achieve higher-throughput confident metabolite identifications. This article discusses recent developments involving the integration of multiple analytical platforms such as ultra-high-performance liquid chromatography (UHPLC)-tandem mass spectrometry (MS/MS)-solid phase extraction (SPE) and nuclear magnetic resonance (NMR) to facilitate higher throughput empirical metabolite identifications. We also explore the potential scope and application of microcrystal electron diffraction (MicroED), a cryogenic electron microscopy (cryoEM) method, to the elucidation of small molecule structures. We believe that the incorporation of MicroED into the UHPLC-MS/MS-SPE workflow might lead to faster and higher-throughput identifications of biologically important unidentified metabolites. Development of these integrated metabolomics platforms can especially benefit the plant and microbial natural products community where large-scale identification of specialized metabolites is a major challenge due to the vast chemical diversity of known as well as truly novel compounds.

## Integrated Analytical Instruments for Confident Metabolite Identifications

Most metabolomics studies over the last decade rely on either MS or NMR with MS being the most widely used technique (Letertre et al., 2021). MS coupled with liquid chromatography (LC) or gas chromatography (GC) has dominated the metabolomics arena due to its increased specificity, sensitivity and relative depth of coverage. Currently, LC-MS offers the greatest depth of coverage and dynamic range for metabolite analyses. Cumulatively, mass spectral database resources are substantially larger compared to NMR databases enabling significantly greater number of metabolite identifications in biological samples. However, NMR offers noninvasive, nondestructive and broad quantification of analytes using a single internal or external standard (Letertre et al., 2021). In addition, the historically lower relative sensitivity of NMR compared to MS has been somewhat mitigated with the advent of newer instruments with higher magnetic field strengths and NMR probes with cryogenically cooled receiver coils. For example, the Bruker 1.7 mm TCI MicroCryoProbe is 14-fold more sensitive than the conventional 5 mm room temperature probes. The increased sensitivity of modern cryoprobes currently enables the detection and identification of nanomole concentrations of metabolites (Molinski, 2010; Bhatia et al., 2019). Although both MS and NMR have their pros and cons, it is clear that none of the current analytical methods offers full coverage of the metabolome. Thus, the combined use of multiple analytical techniques and an integrated metabolomics platform are sensible options to maximize metabolome coverage and facilitate confident metabolite identifications based upon multiple orthogonal datatypes.

According to the Metabolomics Standards Initiative (MSI) guidelines, the confident identity of a metabolite is based upon a minimum of two orthogonal parameters such as retention time, accurate mass, MS/MS fragmentation pattern, collision cross section, NMR chemical shifts etc. relative to an authentic standard (Sumner et al., 2007; Schymanski et al., 2014). The integration of multiple analytical data not only increase the chances of identifying a metabolite, but also increases the level of confidence in the identification, thus potentially reducing false positives in the identification process. A compound identified by accurate mass, MS/MS and further confirmed by NMR has a much higher confidence level compared to a compound putatively or tentatively identified by accurate mass and MS/MS. This strategy of utilizing multiple analytical data from complimentary instruments has been successfully used to confidently identify metabolites in several plants such as tomato, tea and barrel medic (van der Hooft et al., 2011; van der Hooft et al., 2012; Qiu et al., 2016).

Historically, organic chemistry journals require purity, accurate mass/molecular formula and NMR connectivity evidence for the identification of organic molecules. Further, the natural products community utilizes high resolution MS and NMR together for structure elucidation purposes. The development of the hyphenated HPLC-UV-SPE-NMR (Clarkson et al., 2005), HPLC-MS/MS-SPE-NMR (Exarchou et al., 2003; van der Hooft et al., 2011) and UHPLC-MS/MS-SPE-NMR (Sumner et al., 2015) automated purification platforms coupled with structure elucidation/validation by NMR accelerated the traditionally lengthy and laborious natural product discovery process (Letertre et al., 2021). The integration of multiple instruments greatly benefited the metabolomics community as the platform allows automated purification of targeted metabolites from complex biological samples followed by confident identification using a combination of MS/MS, 1D and 2D NMR experiments. Simultaneous purification of multiple targeted metabolites can be achieved by splitting the UHPLC eluent partly towards a MS detector (5%) and partly towards a solid phase extraction (SPE) cartridge (95%) (Bhatia et al., 2019). Repeated injections of the sample lead to the purification and concentration of microgram amounts of desired metabolites within the SPE cartridges that are subsequently eluted using minimal amounts of deuterated solvents for NMR analyses (Bhatia et al., 2019). Important biological spectral features that remain unidentified in a conventional MS or NMR-based metabolomics database search can be identified by analyzing the accurate mass, MS/MS fragmentation patterns and the NMR spectra in a complementary fashion. Additionally, compounds identified through database search can be validated by NMR for a more confident identification. Less than 10 µg of SPE-purified metabolite is reported to be sufficient to generate 1H NMR spectrum suitable for structure validation (van der Hooft et al., 2011). An application of the UHPLC-MS/MS-SPE-NMR integrated platform was demonstrated in the plant metabolomics community by studying the metabolome of the model legume barrel medic (*Medicago truncatula*)*.* Previously, nontargeted metabolic profiling of *M. truncatula* aerial and root tissues by either MS/MS or NMR had resulted in the tentative identification of approximately 40 flavonoids and 80 triterpenoid saponins (Huhman and Sumner, 2002; Kapusta et al., 2005a; Kapusta et al., 2005b; Huhman et al., 2005; Farag et al., 2007; Pollier et al., 2011; Qiu et al., 2016). UHPLC-MS/MS-SPE-NMR confirmed the identity of 88 compounds that are publicly available as part of the Bruker Sumner MetaboBASE® Plant Library (https://www.bruker.com/en/products-and-solutions/mass-spectrometry/ms-software/metabolomics-spectral-libraries.html). The confirmed identity of these compounds was aimed towards elucidation of several gene functions and characterization of triterpene saponin and flavonoid biosynthetic pathways. Overall, UHPLC-MS/MS-SPE-NMR has been proven to be a powerful, cost-effective, and less labor-intensive platform to identify novel metabolites in the model legume.

**FIGURE 1:**
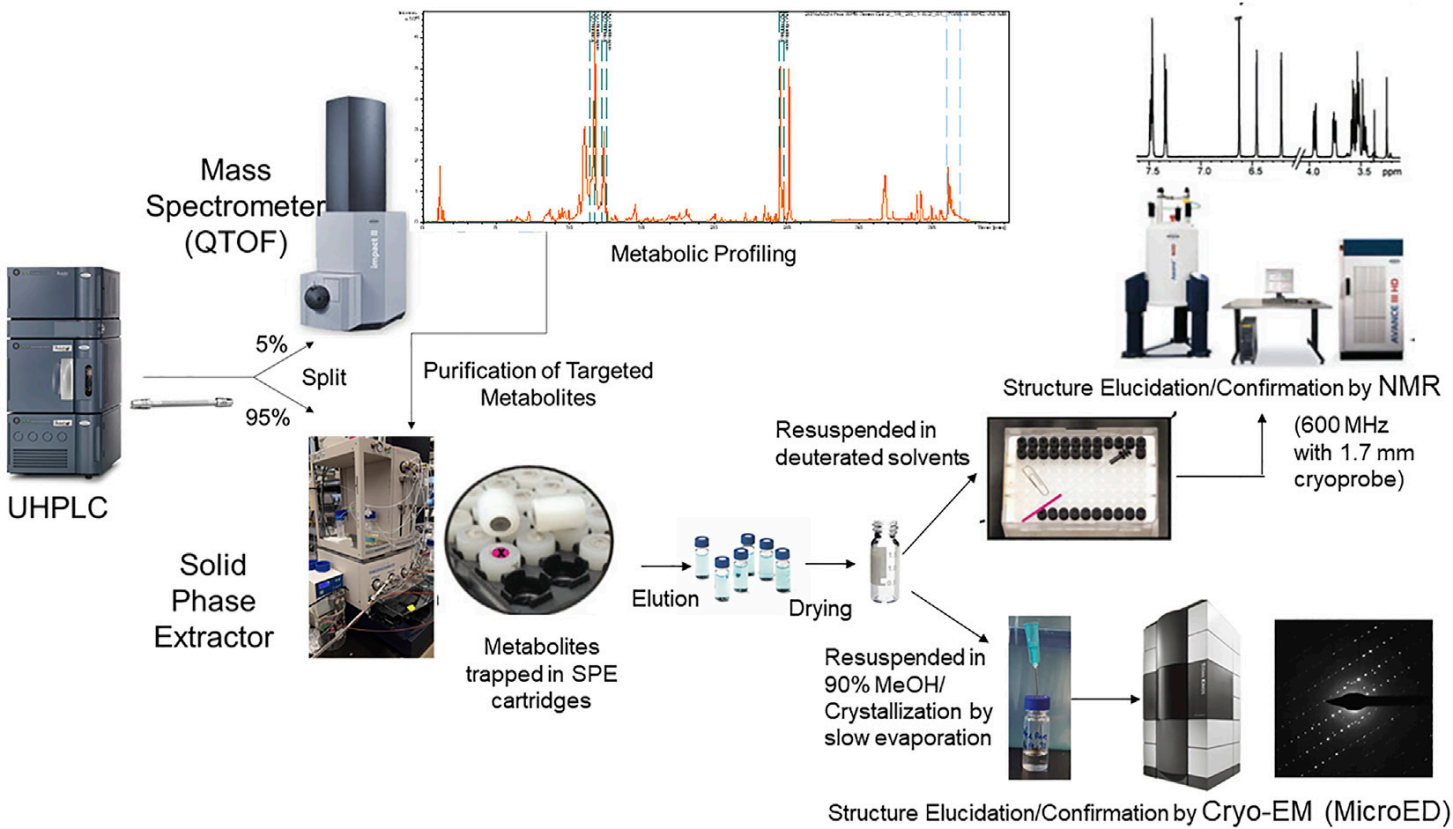
Schematic representation of integrated analytical workflow (UHPLC-MS-SPE-NMR and MicroED) for automated purification and structure elucidation of targeted metabolites. The platform allows putative identification of metabolites based upon retention time, accurate mass, and MS/MS database searches. Targeted metabolites that are unidentified or require further structural confirmation can be purified simultaneously using the UHPLC-MS-SPE system in an automated manner. Structures of purified metabolites can then be confirmed or elucidated by NMR or MicroED.

Absolute structure elucidation or even structure confirmation by NMR can be a lengthy and complex process and its success is often dependent on the NMR interpretation skills of the researcher. Although, substantial progress has been made to simplify compound identification by NMR, it can still be a daunting task for non-experts. In the quest for simpler, faster and higher-throughput confident compound identifications in metabolomics, new technologies such as the cryo-EM method MicroED are being explored. MicroED was recently reported to enable precise stereochemical structure elucidation of milligram quantities of metabolites in a few hours (Jones et al., 2018) and we believe this technology can be pushed further to the structure elucidation of high nanogram to low microgram levels. The addition of MicroED into the existing UHPLC-MS/MS-SPE-NMR platform is exciting as it can potentially pave the way for faster and more straight forward identification of metabolites with less interpretative skills. A schematic representation of integrated analytical instruments (LC-MS-SPE-NMR and MicroED) for automated purification and structure elucidation of targeted metabolites is depicted in Figure 1.

## MicroED in Small Molecule Structure Analyses

In recent years, diffraction-based EM techniques such as MicroED have emerged as an effective tool to elucidate structures of frozen, beam-sensitive samples. MicroED utilizes a cryo-TEM (transmission electron microscope) to collect diffraction data from microcrystalline samples in order to determine their 3D structures (Nannenga and Gonen, 2019; Nannenga, 2020). As electrons interact strongly with matter, MicroED is highly successful for structure determination from crystals several orders of magnitude smaller than those used for single crystal X-ray diffraction. The MicroED method was initially developed for the determination of high-resolution protein structures (Shi et al., 2013; Nannenga BL. et al., 2014; Nannenga B. L. et al., 2014), later used for to the study of peptide structures as well (Rodriguez et al., 2015; Sawaya et al., 2016; Gallagher-Jones et al., 2018; Warmack et al., 2019), and in recent years, MicroED has been extended to the structural study of small organic molecules. The use of MicroED for the high-resolution structure determination of small molecules offers several advantages over other methods of structural analysis. Many structures of organic molecules and materials studied by MicroED have been determined directly from electron diffraction data collected from small amounts of synthesized material, thereby circumventing the need for large amounts of material and time-consuming recrystallization and optimization (Gruene et al., 2018; Jones et al., 2018; Banihashemi et al., 2020; Levine et al., 2020; Gleason et al., 2021). Additionally, MicroED is capable of detecting and solving the structure of several polymorphs or alternative compounds from a single sample preparation (Jones et al., 2018). Because secondary metabolites are often purified in low amounts from their native sources, it can be very difficult to grow large crystals suitable for X-ray diffraction experiments. Therefore, the use of MicroED for the structural studies of natural products is very attractive (Danelius et al., 2021). Previous work has demonstrated the ability of MicroED to determine atomic resolution structures of specialized metabolites, including biotin, niacin, brucine, and thiostrepton, directly from powders obtained following purification or procurement from chemical suppliers (van Genderen et al., 2016; Jones et al., 2018; Zhou et al., 2019). Additionally, MicroED is likely to solve the structures from powder mixtures on the same grid, which was demonstrated using mixtures of biotin, carbamazepine, cinchonine, and brucine powders (Jones et al., 2018). These initial studies indicate that MicroED can be a powerful tool in small molecule structure elucidation and can potentially accelerate compound identification in the natural products and metabolomics community.

## MicroED Structure Analysis Workflow

**FIGURE 2:**
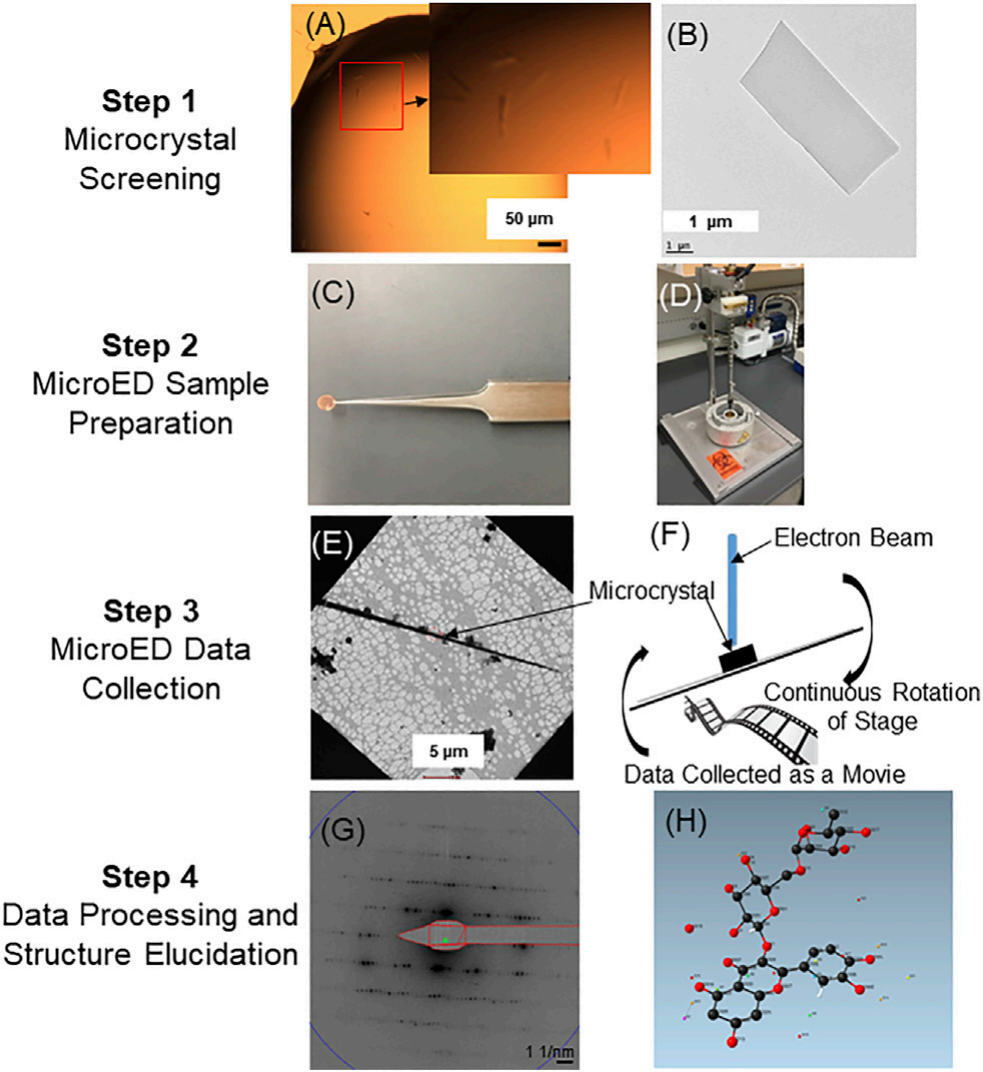
MicroED small molecule structure elucidation workflow. Following purification, samples are initially screened for the presence of microcrystals using either a light microscope or TEM. Panel **(A)** illustrates representative needle-shaped crystals in solution as observed under a light microscope (see zoomed inset of A). Panel **(B)** shows a representative single crystal as observed under a TEM. Once microcrystals are detected, samples (powder or in solution) are applied onto cryo-TEM grids **(C)**. The grids can then be plunge frozen by rapidly submerging into liquid ethane **(D)**. Plunge-frozen grids are then analyzed in the cryo-TEM to detect the presence of well-diffracting single crystals. Panel **(E)** shows the presence of a long needle-shaped microcrystal under low magnification. MicroED data is then collected as a movie from single crystals by continuously rotating the stage as the crystal is exposed in the electron beam **(F)**. Each frame from the continuous rotation diffraction is extracted **(G)** for indexing, integration and scaling by using XDSGUI (Kabsch, 2010). Phase information is then determined by SHELXT (Sheldrick, 2008) followed by refinement using the SHELXLE (Hübschle et al., 2011). Panel **(H)** shows the structure of the flavonoid rutin as determined by MicroED (structure determined from powdered rutin standard purchased from a chemical supplier).

The MicroED sample preparation, data collection, and data processing pipeline for proteins as well as small molecules has been described previously (Shi et al., 2016; Bu and Nannenga, 2021; de la Cruz, 2021; Martynowycz and Gonen, 2021; Zee et al., 2021). A MicroED structure elucidation workflow for natural products and specialized targeted metabolites has been optimized from previously published methods (Figure 2). Samples can be initially screened for the presence of microcrystals using either an optical light microscope or a TEM. Once the presence of microcrystals in the samples are confirmed, they are deposited onto TEM grids by adding powdered samples directly to the grid, creating a crystalline suspension and pipetting the suspension on to the grid followed by blotting or drying, or by solubilizing the sample in a compatible solvent and adding this on the grid followed by drying of the solvent and sample crystallization directly on the grid. Following the deposition of the sample on the grid, the grids are then either frozen in liquid ethane, liquid nitrogen, or loaded into the cryo-TEM at room temperature and allowed to freeze within the cryo-TEM. The grid is then searched at low magnification to identify the presence of crystals on the grid. When a promising crystal has been identified, the crystal is brought to the center of the field of view, which is important as the diffraction mode of the microscope should be aligned such that the beam used for exposure is sampling the same area. After the crystal has been aligned, an initial diffraction pattern is collected to assess the diffracting power of the crystal. If the crystal shows high-quality diffraction (e.g. high-resolution, sharp spots) then a complete dataset is collected. To collect a MicroED dataset, the crystal is first tilted to the maximal angle where no other crystals or grid bars enter the path of the beam. The data collection is then performed by continuously rotating the crystal in the electron beam as the high speed detector records the diffraction data (Nannenga B. L. et al., 2014). It is critical that the stage be at eucentric height so that the crystal does not rotate out of the beam as the stage rotates during data collection. The resulting diffraction movies containing frames of continuous rotation diffraction patterns are converted to Super Marty View (SMV) format to extract each frame (Hattne et al., 2015). The frames are indexed, integrated and scaled using standard diffraction processing programs developed for X-ray crystallography (e.g. XDS (Kabsch, 2010), DIALS (Clabbers et al., 2018)). Datasets, typically consisting of data from several crystals merged together are then phased by direct methods followed by structure refinement with electron scattering factors, using programs commonly used for small molecule structure determination (e.g., SHELX (Sheldrick, 2008)). The structure of the flavonoid rutin as solved by the MicroED structure elucidation workflow is shown in Figure 2.

## Integration of UHPLC-MS-SPE and MicroED

The incorporation of MicroED into the UHPLC-MS-SPE workflow was envisioned to potentially achieve faster, higher throughput and confident metabolite identifications based upon multiple orthogonal data. Similar to UHLC-MS-SPE-NMR, several biologically relevant unidentified or putatively identified metabolites can be targeted and purified simultaneously and further analyzed by MicroED. Preliminary experiments suggest that it is possible to generate sufficient microcrystals of multiple purified flavonoids and triterpenoid saponins from 20 UHPLC-MS-SPE injections of a plant extract (10 mg lyophilized powdered sample extracted with 1 ml 80% methanol). Structure analysis by MicroED requires substantially lesser number of UHPLC-MS-SPE injections compared to NMR where 40–60 plant extract injections are often required to achieve adequate purified material for structure validation by 1D NMR and absolute structure elucidation by 2D NMR. A previous study by Jones et al., 2018 (Jones et al., 2018) reported a 50% success rate of obtaining high quality diffraction data from flash column purified small molecules without additional crystallization. Our initial experiments suggest that slow evaporation of SPE-purified metabolite solutions in 90% methanol at 4°C for 7–10 days has an 80% success rate of producing well-diffracting microcrystals. Ten putatively identified flavonoids and triterpenoid saponins were used to evaluate the crystallization efficiency of the slow evaporation method. Microcrsytals and the corresponding diffraction pattern of SPE-purified 6-malonyl ononin is shown in Supplementary Figure S1. Due to the minute amounts (nanogram-low microgram) of purified metabolites and the resultant low crystal density in our study, crystal screening and identification of high-quality single crystals were often more time-consuming. Automated cryo-EM crystal screening software can potentially accelerate this process in future. It must be noted that the success of MicroED also depends on the quality of microcrystals and further optimization of crystallization conditions is crucial. In our initial studies, we have encountered multiple crystals that are sometimes stacked together leading to low resolution diffraction spots unsuitable for structure determination. Larger and improperly shaped crystals may need to be further broken down by vortexing, sonicating or pipetting. Other alternative and more sophisticated methods for preparation of microcrystals include the use of focused ion beam (FIB) milling under cryogenic conditions (Nannenga and Gonen, 2019). Once suitable microcrystals producing high quality diffraction patterns are found, MicroED data can be acquired in minutes and compounds can be identified in less than an hour. Presence of minor impurities from co-eluting compounds in the crystalline solution do not seem to interfere greatly with structure elucidation of the targeted metabolites. However, improper sample handling can lead to sample degradation and elucidation of only partial structures. This was evident in case of malonylated isoflavonoids where degradation of the highly labile malonyl group was noticed. Overall, in its current form the authors believe that MicroED is best utilized as an orthogonal structure elucidation tool in combination with other orthogonal datasets for higher confident structure determinations. MicroED is not perceived to be a totally independent structural elucidation tool as it still faces challenges in determining bond lengths accurately enough to resolve single from double bonds or alcohols from ketones. As the method continues to develop, it has the potential to be used for absolute structure elucidation of unknown compounds with precise stereochemistry.

For MicroED, there are several areas of active methods development that will continue to push the technique forward. Areas of method development relevant to small molecule structure determination include enhanced data collection procedures, the modeling of charge and chemical bonding within the crystal structures (Yonekura and Maki-Yonekura, 2016; Yonekura et al., 2018), and procedures for using electron diffraction to determine the absolute stereochemistry of a molecule (Brázda et al., 2019). Also, sample preparation is critically important for MicroED and represents an important area of continued development. It is yet to be determined if a simple slow evaporation crystallization strategy is suitable to generate microcrystals for different classes of specialized natural products. Our ongoing efforts are focused on improving the UHPLC-MS-SPE-MicroED workflow for faster, easier and confident metabolite identifications. Several flavonoids, sapogenins and triterpenoid saponins have been SPE-purified and currently being analyzed by MicroED.

## Conclusion

Integrated analytical platforms such as UHPLC-MS-SPE coupled with NMR and MicroED offer alternative and effective approaches toward identification of metabolites that often remain unidentified in conventional metabolomics database search workflows. Introduction of MicroED into the LC-MS-SPE purification and structure elucidation pipeline is especially exciting as it opens up new avenues for faster, easier and higher-throughput identification of metabolites. The most attractive aspect of MicroED is its wide accessibility as electron microscopes and detectors needed for it are available in most modern EM cores. However, it must be noted that wide-spread application of MicroED in small molecule research is relatively recent and further development of automated software packages for crystal screening, data collection and structure determination are still needed for routine analyses. As MicroED becomes more targeted towards small molecule research, we expect to see increased applications of the method in metabolomics workflows.

## Data Availability

The original contributions presented in the study are included in the article/Supplementary Material, further inquiries can be directed to the corresponding author.
