# Supplementary material for: Recent Developments Toward Integrated Metabolomics Technologies (UHPLC-MS-SPE-NMR and MicroED) for Higher-Throughput Confident Metabolite Identifications
Source: Front Mol Biosci. 2021 Sep 2;8:720955. doi: 10.3389/fmolb.2021.720955 (PMC8445028; doi:10.3389/fmolb.2021.720955)
Supplement: Supplementary file 1 [file DataSheet1.docx]

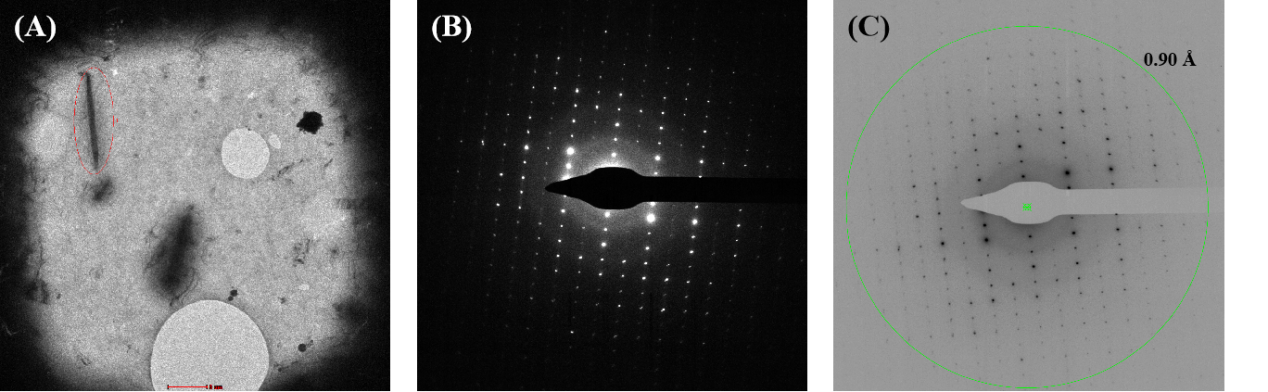


**Supplemental Figure S1.** MicroED data collection of small molecule 6-malonyl ononin. Following purification and MicroED grid preparation, grids were loaded into the cryo-TEM. Panel (A) shows the identification of needle-shape 6-malonyl ononin microcrystal (highlighted in red circle) on the surface of the carbon film, where the scale bar stands for 5 µm. Panel (B) shows a representative MicroED pattern collected on the CETA D detector, where the diffraction spots extended beyond the edge of the detector which is 0.60 Å. Panel (C) shows the pattern (B) processed in XDSGUI (Kabsch, 2010), where the green resolution ring stand for 0.90 Å.
